# Modern challenges of iodine nutrition: vegan and vegetarian diets

**DOI:** 10.3389/fendo.2025.1537208

**Published:** 2025-05-08

**Authors:** Laura Croce, Mario Rotondi, Rosaria Maddalena Ruggeri

**Affiliations:** ^1^ Department of Internal Medicine and Therapeutics, University of Pavia, Pavia, Italy; ^2^ Istituti Clinici Scientifici Maugeri Istituto di ricovero e cura a carattere scientifico (IRCCS), Unit of Endocrinology and Metabolism, Laboratory for Endocrine Disruptors, Pavia, Italy; ^3^ Department of Human Pathology and Childhood “G. Barresi” (DETEV), Endocrine Unit, University of Messina, Messina, Italy

**Keywords:** iodine deficiency, vegetarian diet, vegan diet, iodine supplementation, thyroid

## Abstract

Vegetarian diets are gaining popularity worldwide, especially among women and in the younger part of the population, rising some concerns for the risk of inadequate iodine requirements if the diet is not correctly planned. Indeed, subjects under vegetarian dietary regimens, mainly the vegan ones, are at risk of developing both iodine deficiency and excess, due to lack of animal-derived foods on the one hand, and to the use of vegan alternatives (such as seaweed) and over-the-counter supplements on the other hand. Women in childbearing age and children are particularly vulnerable to the adverse thyroid consequences. Thus, this paper aims to provide some practical information to achieve adequate iodine intake and avoid adverse effects on thyroid in this population.

## Introduction

In recent years there is growing awareness of the potential benefits of plant-based diets, and vegetarian diets, that avoid some or all foods of animal origin, have gained in popularity, so that increasing number of individuals at any age is turning to vegetarianism supported by ethical/religious and ecological motivations, health concerns, and taste preferences (1, 2).

The umbrella term “vegetarianism” covers a range of dietary choices, whose common distinctive features are: i.) consumption of a wide variety of plant foods; ii.) Exclusion of flesh foods: all types of meat, meat products (sausages, salami, etc.), and fish (including sushi, mollusks and crustaceans). There are two main types of vegetarian diet: the lacto-ovo-vegetarianism (LOV), that includes dairy products, eggs, and honey (further subdivided in the subcategories lacto-vegetarianism which excludes eggs, and ovo-vegetarianism which excludes dairy products); and the veganism, that excludes animal foods at all (3, 4). There are also growing numbers of people in Western countries that have chosen to reduce their animal product consumption, and adopted a plant-base diet, ranging from pescatarian (excluding meat/poultry, but including fish/seafood) regimens, to demi-vegetarian (or flexitarian) that occasionally include meat/poultry/fish (less than once a week) (2). Consequently, the nutritional profiles of vegetarian and plant-base diets can vary considerably. Compared with omnivorous diets, vegetarian diets contain more folate, fibers, antioxidants, sterols, phytochemicals and carotenoids, and less saturated fatty acids, animal protein and cholesterol, but may be weak in some minerals, such as iron, calcium and iodine, and vitamins (mainly B12) from animal source foods, if not careful planned and well balanced according to current guidelines (2, 5).

Moreover, the rising demand for exclusively plant-based products has increased the commercial offer for alternative products, such as plant-based milk- and meat-substitutes, but also highly processed, ready-to-eat foods (6). Although the variety of foods available for vegetarians has increased in the last years, and most commercial plant-based foods are enriched in vitamins and micronutrients (iron, calcium, B12, or vitamin D), there remain some concerns as for the difficulties in reaching adequate amounts of iodine (7). The risk of iodine shortfall is of particular concern in children and pregnant/breastfeeding women.

## Iodine nutrition and vegan/vegetarian diets

Iodine is a rate-limiting element for the synthesis of thyroid hormones, and it becomes a determinant of thyroid disease when not adequately supplied. This trace element can be obtained exclusively through the diet or through iodine supplements, and cannot be replaced by any other nutrient. Iodine is naturally present in some foods, especially milk and seafood, mainly as an iodide salt, but also in other chemical forms, including iodate, molecular iodine or monoatomic iodine incorporated in proteins. Iodide is almost completely absorbed in the stomach and duodenum, and enters the circulation. The thyroid gland concentrates it in appropriate amounts for thyroid hormone synthesis due to a specific iodide-sodium symporter (NIS), expressed on thyroid cell surface, and most of the remaining amount is excreted in the urine (8). Urinary iodine reflects dietary intake directly because more than 90% of dietary iodine is excreted in the urine (9). Median urinary iodine concentrations of 100–199 μg/L in children and adults, 150–249 μg/L in pregnant women and >100 μg/L in lactating women indicate adequate iodine intakes (10). The Recommended Dietary Allowances (RDA) of iodine are 150 μg/day for adults and 250 μg for pregnant/lactating women, according to the World Health Organization (WHO) (11) (**Table 1**). Similarly, dietary reference values have been provided by the European Food Safety Authority (EFSA), indicating as average requirement (AR) 150 µg/day for adults, 70-130 µg/day for infants aged 7–11 months and for children, and 200 µg/day for pregnant women. A value of 600 µg/day was also proposed as a Tolerable Upper Intake Level (UL) for adults, including pregnant and lactating women (12).

**Table 1:** Iodine recommended intakes and nutritional status evaluation in different populations.

IODINE NUTRITION STATUS ASSESSMENT BY UIC		
Children and adults		
<20 μg/L	Insufficient	Severe ID
20–49 μg/L	Insufficient	Moderate ID
50–99 μg/L	Insufficient	Mild ID
100–299 μg/L	Adequate	Sufficient
**≥300 μg/L**	**Excessive**	**Excess iodine**
Pregnancy		
<150 μg/L	Insufficient	ID
150–249 μg/L	Adequate	Sufficient
**≥500 μg/L**	**Excessive**	**Excess iodine**
Lactation		
<100 μg/L	Insufficient	ID
> 100 μg/L	Adequate	Sufficient
**≥500 μg/L**	**Excessive**	**Excess iodine**
RECOMMENDED DIETARY ALLOWANCES (RDAs) FOR IODINE		
Birth to 6 months	110 μg/day	
7–12 months	130 μg /day	
1–8 years	90 μg/day	
9–13 years	120 μg/day	
14–19+ years	150 μg/day	
Pregnancy and Lactation	250 μg/day	
TOLERABLE UPPER INTAKE LEVELS (ULS) FOR IODINE		
1–3 years	200 μg/day	
4–8 years	300 μg/day	
9–13 years	600 μg/day	
14–18 years	900 μg/day	
19+ years	1100 μg/day *	

ID, iodine deficiency; UIC, urinary iodine concentrations. In bold the upper thresholds of UIC for iodine excess.

*Pertinent references:

World Health Organization. United Nations Children’s Fund & International Council for the Control of Iodine Deficiency Disorders. Assessment of iodine deficiency disorders and monitoring their elimination. 3rd ed. Geneva, Switzerland: WHO, 2007 (10).

Institute of Medicine, Food and Nutrition Board. Dietary Reference Intakes for Vitamin A, Vitamin K, Arsenic, Boron, Chromium, Copper, Iodine, Iron, Manganese, Molybdenum, Nickel, Silicon, Vanadium, and Zincexternal link disclaimer. Washington, DC: National Academy Press, 2001 (53).

EFSA Panel on Dietetic Products, Nutrition and Allergies (NDA). Scientific Opinion on Dietary Reference Values for iodine. EFSA Journal 2014; https://doi.org/10.2903/j.efsa.2014.3660 (12).

https://sinu.it/wp-content/uploads/2025/01/APPENDICI_abcd.pdf (61).

Leung AM, Avram AM, Brenner AV, et al. Potential risks of excess iodine ingestion and exposure: statement by the American Thyroid Association public health committee. Thyroid. 2015;25(2):145-146 (54).

*The American Thyroid Association advises against 500 µg iodine daily for both children and adults and during pregnancy and lactation^2^.

The iodine content of foods is highly variable between food categories as well as within each category, as shown in **Table 2**. Good food sources of iodine include fish and other seafood as well as eggs and milk, as well as their derivatives, given that iodine content of milk and eggs is influenced by animal feeding and hygienic practices. Meat, animal products and dairy products, contain iodine in a variable amount depending on whether farm animals have received iodine fortified feeds. Anyway, milk represents a relevant source of iodine, mainly in children (13), whereas plant-based beverage used as milk substitutes, such as soy and almond beverages, contain relatively small amounts of iodine unless fortified (14). Most fruits and vegetables contain little amounts of iodine, depending on the iodine content of the soil, and fertilizer use. Similarly, cereals and bread are poor food sources of iodine unless fortified (15). Despite seaweed (such as kelp, nori, kombu, and wakame) are among the best food sources of iodine, the iodine amounts in different seaweed species vary greatly, and may result in iodine excess. Iodine can be added to salt, and it is accepted that 30 ppm (30 mg of potassium iodate per kilogram of salt) is the lowest level that will ensure the provision of 100 μg of iodine per day (10). Iodine fortification of salt has been implemented in several countries on a mandatory or voluntary basis. Finally, iodine is available as a dietary supplement, and its content must not exceed 225 μg daily. A supplement of 150 μg/day iodine in the form of potassium iodide is advisable in subjects at risk of inadequate iodine intake (11).

**Table 2:** Iodine content in common foods.

Description	Iodine (mcg/ serving)	LactoOvo-Vegetarian	Vegan
Baked Cod	146.3		
Nori seaweed, dried	115.8		
Nonfat Yogurt*	100.1		
Whole Milk *	81.8		
Iodized Salt	78.2		
Fish sticks, oven-cooked	56.8		
Eggs, hard-boiled*	30.5		
Pasta, boiled in water with iodized salt	29.6		
Fresh bluefin tuna, cooked	19.6		
Mozzarella*	15.3		
Shrimps	12.9		
Salmon fillets, baked	10.9		
Canned tuna	7.4		
Veggie burger, soy based, baked	5.3		
Parmesan Cheese *	4.1		
Spinach, boiled	3.5		
Beef roast	3.3		
Soy Beverage	3.2		
Lamb chop, pan-cooked	2.1		
White bread *	1.2		
Chicken breast, roasted	1.0		
Butter	0.6		
Broccoli, boiled	0.4		
Cucumber	0.4		
Peach	0.3		
Rice, cooked	0.3		
Margarine	0.2		
Tomato	0.2		
Soy sauce	0.1		
Apple	0.1		
Avocado	0.1		
Lettuce	0.1		
Corn flakes	0.1		
Sea salt, non-iodized	0.02		
Olive Oil	0		
Potato, boiled	0		
Beans, boiled	0		
Lentils, cooked	0		
Tofu	0		
Almonds	0		

*Iodine content can vary based on country-specific iodine food enrichment policies, iodine concentration in soil and in fodder.

Green color indicates foods included in LactoOvo-Vegetarian and Vegan Diets. Adapted from the USDA, FDA and ODS-NIH Database for the Iodine Content of Common Foods per Serving, Release 4, October 2024 (https://www.ars.usda.gov/ARSUserFiles/80400535/Data/Iodine/IODINE_DATABASE_RELEASE_4_PER_SERVING.pdf).

A U-shaped relationship exists between iodine nutritional status and thyroid disorders, indicating that either too little or too much iodine may be harmful to the gland (16). The interval between inadequate and a more-than-adequate iodine intake is relatively narrow, so that even small changes may have profound effects on the prevalence of thyroid dysfunction and goiter (17). Iodine deficiency (ID), defined as a median urinary iodine concentration (UIC) <100 μg/L, is a major cause of hypothyroidism worldwide, mainly when severe (UIC <20 μg/L) or moderate (UIC <50 μg/L), and it also favours the development of goitre and thyroid nodules (18). Nevertheless, also an excessive iodine intake, due to inappropriate supplementation or excess intake of iodine-rich food, can be harmful to the thyroid, increasing the risk for thyrotoxicosis, hypothyroidism and autoimmunity (16, 17).

In general, an adequate iodine supplementation, reaching a population median UIC between 100 and 300 μg/L, is considered safe, with the benefits largely outweighing the potential risks. Universal salt iodization (USI) represents the best and safest strategy to implement iodine intake and prevent/eradicate ID in the general population, according to the WHO (10).

Nowadays in most industrialized countries, the main sources of iodine are iodized salt, fortified foods (such as bread), and animal-derived foods (cow milk, dairy products, fish, seafood, eggs) (19), while plant-derived foods contain low-quantities of iodine, unless fortified, with the only exception of seaweed (20). In addition to iodized salt and seafood, milk and dairy products are among the major sources of iodine in Mediterranean and Western diets, due to supplementation of iodine in animal feed (21).

As vegetarian diets are becoming more and more popular worldwide, especially among women and young people, concerns for iodine nutrition have raised (22). Indeed, plant-based foods have a lower iodine content than animal-derived ones, and vegan diets exclude many iodine-rich foods, including dairy, eggs, and/or fish (7). Since cow milk has demonstrated a significant role in contributing to the total iodine intake, especially in children, as demonstrated in UK and Norway-based studies, vegan diets eliminating milk-derived food are particularly at risk for iodine deficiency (23). Several studies have highlighted that subjects under vegan diet, who strongly restrict consumption of iodine-rich foods and are dependent only on iodine deriving from plants are at high risk of iodine deficiency (24). Iodine nutrition has been demonstrated to be inadequate among vegetarians, when assessed both as median UIC (25–28) and estimated iodine intake (25–29), and is particularly poor among vegans. Iodine intake in vegans appears to be even lower among women and in countries lacking USI programs, compared to countries in which iodine intake derives mainly from iodized salt or fortification of bread (30). Some studies also suggest that both vegetarians and vegans, especially women, also tend to consume less iodized salt (31). A further risk for vegetarians would stem from the combined risk of having also iron and selenium deficiency, which would increase the detrimental effect of iodine deficiency on thyroid function (25). For this reason, the most important Scientific Societies of Nutrition and Vegetarian/Vegan Nutrition recommend iodized salt and/or iodine containing supplements (150 μg/d) in childbearing age vegan women who do not consume iodized salt (28). Moreover, a LOV diet, that include milk, dairy products and eggs, provide adequate iodine sources, given that iodized salt is always recommended (23).

Finally, in vegetarian diets, an alternative source of iodine is represented by seaweeds (such as macroalgae,nori and kelp). However, even seaweed has a highly variable content of iodine, according to the species and processing of food, and can even expose users to iodine excess, so that uncontrolled consumption of seaweed should be discouraged (32).

## Nutritional advices for mothers and children

Iodine nutrition is particularly crucial in women in fertile age, since during pregnancy ID can cause an increased risk of growth retardation and brain damage for the fetus, and induce both maternal and fetal goiter (18, 33, 34). The iodine requirements increase during pregnancy, due both to an increased maternal thyroid hormone synthesis, greater urinary iodine loss due to an increased glomerular filtration rate and iodine transfer to the fetus (35). Several studies suggest that pregnant and lactating women may be at high risk of having inadequate iodine levels, even in countries where salt iodization programs are available (36, 37). Since iodized salt may not be a sufficient source of iodine to meet the minimum requirements of this vulnerable group, several international authorities recommend a daily supplement of 150 μg of iodine for pregnant and lactating women (38). This appears even more relevant since childbearing age women are generally more likely to follow a plant-based diet, especially in the 24–39 years age bracket (39). A recent Australian study specifically aimed at assessing iodine nutrition in women of childbearing age showed that vegan women had lower UIC and iodine intake when compared to omnivores, even if both groups had median UIC below the threshold for iodine deficiency (27).

Another recent Australian study highlighted that pregnant women following a vegetarian diet were more likely to report multivitamin use, even if urinary iodine levels were similar between multivitamin users and non-users (40).

It should be noted that a recent case report described a case of transient hypothyroidism with goiter due to severe ID in the child of a vegan mother with a severely restricted iodine intake from the UK (41). Nevertheless, studies regarding the possible impact of iodine deficiency in vegetarian/vegan mothers on pregnancy outcomes are lacking. Indeed, several studies show that a plant-based diet could reduce the risk of several pregnancy complications, such as pre-eclampsia, gestational diabetes and pre-term birth. In conclusion, vegetable based diets can be safe during pregnancy, but they may require a dedicated nutritional intervention to provide a balanced intake of key nutrients, including iodine, and to prevent maternal undernutrition (42).

Also breastfeeding mothers have an increased requirement of iodine, and some recent evidence suggest that vegan mothers could be at risk of not providing enough iodine to their children with breast milk. A recent study performed on lactating women showed that the breast milk iodine content was lower in a small sample of vegan and vegetarian mothers when compared with omnivorous ones, and that most samples indicated an inadequate iodine content (43). A comparison of the micronutrient composition of breastmilk of vegan mothers compared to that of omnivorous breast milk donors showed lower iodine content, even if in the normal range, in the vegan group. UIC was lower in vegan mothers when compared with omnivorous ones, even if the estimated iodine intake was similar (44). Nevertheless, a recent study correlating the maternal dietary choices to the iodine status of the offspring did not find any correlation between vegetarian practice and whether or not the mothers included dairy products in their diets and the toddlers UIC (45).

The risk of ID due to a plant-based diet is relevant also in children, since in vegetarian/vegan households children’s eating behavior depends on parents, who often share eating habits setting an example and wanting their children to share their beliefs. Moreover, several establishments such as kindergartens, colleges, schools and hospitals are providing vegetarian options for children for educational, health and environmental reasons. While plant-based diets provide a protection towards non-communicable diseases such as cardiovascular ones, initial data suggest that they could cause stunted growth (especially among vegan children) and alterations in body composition (46). In this context, data on iodine nutrition in vegetarian children are scarce. A recent German study found that both vegan and vegetarian children had lower iodine intake when compared to omnivores (47). On the contrary, a similar UIC was found by a Finnish study among vegan children when compared to omnivores (48). A recent study from the Czech Republic showed a trend towards lower UIC values in vegetarians and vegan children when compared to omnivores, even if median values were above the threshold for defining ID in all dietary groups. Vegan and vegetarian children also had a higher rate of AbTg positivity (49). Another possible source of concern is the widespread use of plant-based substitutes of cow milk for vegetarian and vegan newborns and toddlers: these drinks are usually not fortified with iodine and almost completely lack in this nutrient (50). Moreover, seaweed consumption is generally contraindicated in children, due to a higher risk of iodine overload and heavy metals contamination (51).

Given the high risk of ID in children following a plant-based diets, it seems reasonable to suggest the use of iodized salt with a varying amount according to fortification regimens (52). In vegetarian/vegan women, a reasonable approach would be to start low-dose iodine supplements optimally 3 months prior to pregnancy, and to consume iodized salt adding an oral supplement of 150 – 225  μg per day of iodine, in the form of potassium iodide during pregnancy and breastfeeding (53).

## The supplement paradox: more isn’t always better

Paradoxically, subjects under vegan and vegetarian diet are also at risk of developing complications from excess iodine, since vegan alternatives, such as seaweed, contain an excess amount of iodine that may disrupt thyroid homeostasis. Indeed, the availability of iodine from seaweed is largely variable, and it can provide amounts up to a hundred times the RDAs (54). Furthermore, dietary supplements marketed to improve iodine nutrition and thyroid function in vegan and vegetarians, often contain iodine and kelp products in amounts that largely exceed not only the RDA (150 µg per day in adults) but also the daily tolerable upper limit for iodine (TUL, 1000 µg per day) (55). An even lower threshold for safe iodine intake has been proposed by EFSA (600 µg iodine daily) (12) and by the American Thyroid Association (ATA) (500 µg iodine daily) (56). A recent review by Farebrother et al., showing data from a survey of U.S. multivitamin supplements, found significant discordance between label information and laboratory assay, most products containing excess iodine from kelp. These easily accessible dietary supplements potentially expose users to the risk of thyroid dysfunction (57). People should be aware that products deemed as ‘‘natural’’ are not automatically healthy and safe, and many iodine supplements contain kelp, thus providing excess amounts. Mostly, children and pregnant and breastfeeding women are more susceptible to adverse effects of excess iodine, as well as the elderly and individuals with preexisting thyroid diseases. For this reason, the American Thyroid Association (ATA) advises against the ingestion of iodine and kelp supplements containing in excess of 500 µg iodine daily for both children and adults and during pregnancy and lactation (56).

As a source of iodine, vegetarians should use iodized salt, and, if convenient, supplements containing potassium iodide in adequate amounts (150 µg/day). As regarding the use of iodized salt, it should be mentioned that concerns exist on the cardiovascular risks deriving form excess salt consumption. There is evidence of a causal relationship between sodium intake and blood pressure, and excessive sodium consumption (>5 g sodium per day, e.g. one small teaspoon of salt per day) has been shown to be associated with an increased prevalence of hypertension with age (58). Accordingly, the 2020 International Society of Hypertension Global Hypertension Practice Guidelines (59), the 2018 European Society of Cardiology Hypertension Guidelines (60) and the WHO 2023 statement (61) recommend that sodium intake should be limited to <2 g per day (equivalent to <5 g salt per day) in the general population as well as in hypertensive patients. Beyond reducing the quantity of salt added as above reported, they recommended to limit the consumption of high salt containing foods, such as fast foods, soy sauce and processed foods, encouraging consumption of fresh vegetables and fruits. It is also worth of mention that, currently, there is no convincing evidence, from randomized controlled trials, that low salt intake (<2 g/day) is more effective than moderate intake (<5 g/day) in terms of reduction of cardiovascular risks and adverse outcomes (62). Thus, adding no more than 5 grams/d of iodized salt to foods provides 150 ug/die of iodine (that is the RDA) without increasing the risk of hypertension due to excess sodium intake (3, 63).

The recommended use of added iodized salt (no more than 5 gr/day) is useful to the thyroid function and safe with regard to the risk of hypertension and related CVD. Thus, it should not represent a limitation to promote iodine supplementation in the general population (63). We suggest iodized salt to ensure iodine sufficiency both in vegetarians/vegans and in subjects following a Mediterranean diet. In vegetarian/vegan subjects, iodine nutrition should be improved also by assuming pharmacological supplements with a titrated dose of 150 µg/day, and by using enriched foods (see **Table 2** for nutritional content of iodine in foods and vegan alternatives). Also iodine-exclusive supplements deriving from algae (mainly kelp) with a titrated dose (200-250 µg) of iodine are available, but the use of kelp products should be discouraged, according to the ATA recommendations (56). Also, they cautioned against taking iodine supplements with more than 150 μg in a daily dose, that represents RDA for adults.

## Conclusions

**Figure 1:**
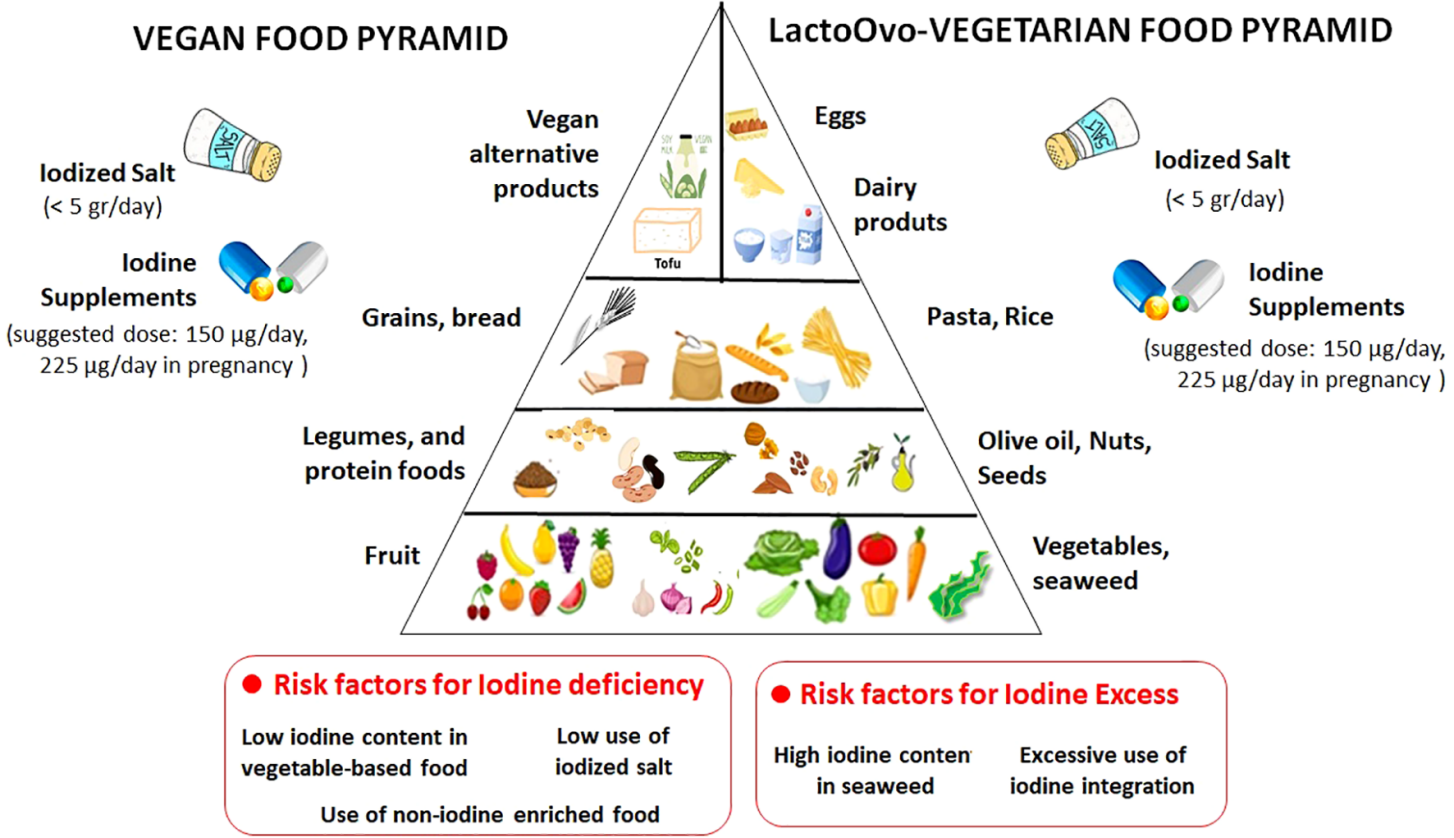
Food pyramid including the typical food groups from Lacto-Ovo (LO)-Vegetarian and Vegan Diet, and possible risk factors for inadequate or excess iodine intake. A well planned and balanced dietary approach can ensure optimum nutrition and well-being. Be aware of having to: - Eat a wide variety of foods from each food group. Variety helps ensure you consume sufficient quantities of a broad range of nutrients, phytochemicals, and fiber. -Drink enough water to stay hydrated. - Use iodized salt and/or use a mineral supplement that provides 150 μg of iodine, to achieve an adequate and safe iodine intake.

In conclusion, a well-planned vegetarian diet can provide health benefits while aligning with ethical and environmental principles, provided that potential nutritional inadequacies should be taken into account. A healthy and nutritionally adequate vegetarian diet can be obtained including a wide variety of plant foods traditionally consumed in Mediterranean diets (cereals, pulses, vegetables, fruits, seeds, nuts, olive oil), and the necessary supplements (for instance, a reliable source of vitamin B12). Iodine nutrition is of concern since vegan diet are at risk of ID and guidelines on vegetarian/vegan diets often provide neutral advice on supplementing certain nutrients with plant sources without mentioning any potential risks. Iodized salt represents an effective, safe and inexpensive tool to improve iodine nutrition and it should be recommended in subjects under vegetarian regimens of any grade and at any age, as well in subjects following a Mediterranean diet. To further implement iodine intake, vegans should use iodine-fortified plant-based milk alternatives, while consumption of sea vegetables should be discouraged, to prevent excess iodine intake. A daily supplement containing 150 μg of iodine could be suggested, mainly to childbearing women (**Figure 1**). Improved awareness of iodine sources to people under vegetarian/vegan diets is necessary to avoid both inadequate iodine intake in those who do not use iodized salt and/or supplements, and excess iodine intake in over-the-counter supplements and/or seaweed users. In any case, a nutritional counselling is essential to guarantee an adequate iodine intake while planning a vegetarian/vegan diet, particularly among the most susceptible population groups.
